# Cofilin-1 Inactivation Leads to Proteinuria – Studies in Zebrafish, Mice and Humans

**DOI:** 10.1371/journal.pone.0012626

**Published:** 2010-09-08

**Authors:** Sharon Ashworth, Beina Teng, Jessica Kaufeld, Emily Miller, Irini Tossidou, Christoph Englert, Frank Bollig, Lynne Staggs, Ian S. D. Roberts, Joon-Keun Park, Hermann Haller, Mario Schiffer

**Affiliations:** 1 Department of Biochemistry, Microbiology and Molecular Biology and the School of Biology and Ecology, University of Maine, Orono, Maine, United States of America; 2 Department of Medicine/Nephrology, Hannover Medical School, Hannover, Germany; 3 Mount Desert Island Biological Laboratories, Salsbury Cove, Maine, United States of America; 4 Leibniz Institute for Age Research-Fritz Lipmann Institute e.V. (FLI), Jena, Germany; 5 Department of Cellular Pathology, John Radcliffe Hospital, Oxford, United Kingdom; University of Birmingham, United Kingdom

## Abstract

**Background:**

Podocytes are highly specialized epithelial cells on the visceral side of the glomerulus. Their interdigitating primary and secondary foot processes contain an actin based contractile apparatus that can adjust to changes in the glomerular perfusion pressure. Thus, the dynamic regulation of actin bundles in the foot processes is critical for maintenance of a well functioning glomerular filtration barrier. Since the actin binding protein, cofilin-1, plays a significant role in the regulation of actin dynamics, we examined its role in podocytes to determine the impact of cofilin-1 dysfunction on glomerular filtration.

**Methods and Findings:**

We evaluated zebrafish pronephros function by dextran clearance and structure by TEM in cofilin-1 morphant and mutant zebrafish and we found that cofilin-1 deficiency led to foot process effacement and proteinuria. *In vitro* studies in murine and human podocytes revealed that PMA stimulation induced activation of cofilin-1, whereas treatment with TGF-β resulted in cofilin-1 inactivation. Silencing of cofilin-1 led to an accumulation of F-actin fibers and significantly decreased podocyte migration ability. When we analyzed normal and diseased murine and human glomerular tissues to determine cofilin-1 localization and activity in podocytes, we found that in normal kidney tissues unphosphorylated, active cofilin-1 was distributed throughout the cell. However, in glomerular diseases that affect podocytes, cofilin-1 was inactivated by phosphorylation and observed in the nucleus.

**Conclusions:**

Based on these *in vitro* and *in vivo* studies we concluded cofilin-1 is an essential regulator for actin filament recycling that is required for the dynamic nature of podocyte foot processes. Therefore, we describe a novel pathomechanism of proteinuria development.

## Introduction

Podocytes are highly specialized epithelial cells covering the visceral side of the glomerular capillary. They consist of a cell body that gives rise to major processes and minor foot processes (FPs) that are anchored to the glomerular basement membrane via integrins at the focal contact sites. The FPs of neighboring cells form a branched interdigitating network and are interconnected by a multiprotein complex that covers the spaces between the FPs and the slit diaphragms (SD) [1]. The SD is the final barrier for high molecular weight molecules preventing their loss in the glomerular filtrate. The focal contacts as well as the SD are functionally linked to the actin cytoskeletal backbone of the foot process, making actin the common denominator of podocyte function [2].

Podocyte foot process effacement is commonly observed in a large variety of glomerular diseases associated with proteinuria [3]. The pathophysiological basis of podocyte effacement is the reorganization of the actin cytoskeletal backbone of the foot process. The characteristic parallel contractile actin bundles found in the podocyte reorganize into dense actin networks which results in loss of the normal interdigitating pattern, dysfunctional filtration slits and the development of proteinuria [4]. Since foot process effacement is a reversible process, it is critical to understand the molecular mechanisms underlying these structural changes and effacement as well as recovery would require an orchestrated reorganization of actin filaments [5]. A variety of genetic alterations of podocyte proteins indirectly lead to an aberrant cytoskeletal structure, indicating a sensitive signaling balance that can be influenced by mechanical as well as cytokine based signals [6]–[9]. Thus far, mutations in the α-actinin-4 gene are the only known genetic alterations related to the actin cytoskeleton that lead to a podocyte phenotype similar to progressive glomerulosclerosis in humans. The five identified, distinct point mutations in the head-domains of the α-actinin protein, all mediate increased actin cross-linking [10]–[12]. In this study we wanted to examine the role of the actin depolymerizing factor (ADF)/cofilin (AC) family of proteins in podocytes. The AC family of proteins binds both globular (G-Actin) and filamentous actin (F-actin) and this binding is regulated by multiple mechanisms: phosphorylation, interaction with polyphosphoinositides, pH, and competitive binding interactions with other actin binding proteins [13], [14]. When cofilin becomes phosphorylated by the kinases, LIMK1/2 or TESK, on serine 3, cofilin can no longer bind F-actin or G-actin [15]. The phosphatases, slingshot and chronophin, dephosphorylate and activate cofilin [16]. These regulatory elements strictly control the ability of cofilin to bind actin through upstream signaling cascades in response to activation of receptor tyrosine kinases (RTKs) and integrins and through regulating each other [14]. Once activated, cofilin at lower concentrations binds to F-actin and induces a twist in the filament which weakens the actin-to-actin attachments and results in filament severing. At higher cofilin concentrations, cofilin decorates the filaments and appears to enhance P_i_ release from actin monomers within the filament, to increase actin treadmilling and actin depolymerization from the filament pointed end [17].

In this manuscript we found that cofilin was required for normal glomerular filtration in morphant and mutant zebrafish. We demonstrated uncharacteristic actin reorganization and podocyte migration behavior *in vitro* in the absence of cofilin-1 and we found aberrant cofilin-1 activation in diseased human tissues.

## Methods

### Ethics Statement

Animal work was conducted according to the guidelines of the American Physiologic Society and was approved by Institutional Animal Care and Use Committee of the Mount Desert Island Biological Laboratory, Maine (IACUC protocol #0804). All efforts were made to minimize the number of animals used and their suffering.

### Clinical renal biopsy samples

Renal tissue was obtained from the archives of the Department of Cellular Pathology, John Radcliffe Hospital, Oxford. Paraffin-embedded specimens of normal kidney from nephrectomies performed for tumour, and renal biopsy samples from adults with primary focal segmental glomerulosclerosis (FSGS), minimal change disease (MCD) and idiopathic membranous nephropathy (MN) were included in this study. All patients with renal disease had nephrotic-range proteinuria at the time of biopsy. Ethics approval was obtained from the Oxford Research Ethics Committee B (Reference #C02.062) for use of human tissue. All patients gave written consent at hospital admission to experiments on their anonymized archived tissue samples.

### Zebrafish stocks, injections and functional assays

Zebrafish (AB) were mated and grown at 28.5°C in embryo rearing media (E3) as previously described in [18]. Using the Nanoject II injection device (Drummond Scientific, Broomall, PA), cofilin-1 and scrambled control morpholinos were injected into one to four-cell stage fertilized embryos at 100 µM concentrations in 4.6 nl injection buffer (20 mM Hepes, 200 mM KCl and 0.01% phenol red). Morpholino sequences were designed and ordered from GeneTools (Philomath, OR) as follows: standard control sequence - 5′-CCTCTTACCTCAGTTACAATTTATA-3′ and cofilin-1 sequence – 5′-TTGAATGAGCTGTGATGCAGTCCCT-3′. For glomerular function studies, 48 hours post fertilization (hpf) dechorionated embryos were anesthetized with a 1∶20 to 1∶100 dilution of 4 mg/ml Tricaine (MESAB: ethyl-3-aminobenzoate, methanesulfonate acid salt, 1% Na_2_HPO_4_, pH 7.0) (Sigma-Aldrich) and placed in a 1.2% agarose mold for injection. The embryos were then injected either with 70-kDa rhodamine-labeled or FITC-labeled dextrans into the cardiac venous sinus (Molecular Probes, Eugene, OR). Immediately after injection, the embryos were placed in 200 µl ERM in single wells of 96 well plates and allowed to recover. For observation of rhodamine-labeled dextran passing the glomerular barrier, we used the Wt1b-GFP transgenic zebrafish line [19] which was a gift from Christoph Englert, Jena. Fluorescent FITC-dextran clearance from the pupil of the embryo eye was measured in AB-wildtype embryos and evaluated as previously described in Hentschel et al. [18]. The animal protocol was approved by the Mount Desert Island Biological Laboratory (MDIBL) Animal Care Committee. Cofilin-1 mutant embryos, obtained from the Zebrafish International Research Center (ZIRC), were the result of mating wildtype AB female zebrafish with sperm from an AB zebrafish heterozygous for the *hi3736aTg* proviral insertion in the *cfl1* gene [20]. These embryos were grown to six months of age and then mated with AB wildtype zebrafish in the University of Maine zebrafish facility (IACUC protocol #A2008-2-07). Heterozygous zebrafish resulting from this mating were confirmed by PCR and mated to spawn cofilin-1 mutant fish.

### Transmission Electron Microscopy (TEM)

Both morphant and mutant larval zebrafish were sampled at 120 hpf and fixed in 1.5% glutaraldehyde/1% PFA and 70 mM NaPO_4_, pH 7.2. After fixation the embryos were washed three times in 0.2 M cacodylate buffer and then postfixed in 1% osmium tetroxide for one hour at room temperature. After rinsing with cacodylate buffer all specimens were dehydrated in a graded ethanol series and infiltrated and embedded with epon according to manufacturer's protocol (Hard Plus Resin 812, Electronmicroscopy Sciences, Hatfield, PA). Ultra-thin (80–100 nm, thick) sections of the kidney were cut using a Leica Rotary Microtome and mounted on slot and 300 mesh grids (Luxel, Friday Harbor, WA). The sections were stained with 2% uranyl acetate in distilled water, and contrasted with lead citrate. Sections were viewed and photographed on a JEOL-1230 Transmission Electron Microscope (Eching,Germany).

### Podocyte Culture

Murine podocytes were cultured in RPMI-1640 cell medium (Biochrom) containing 10% FBS, penicillin/streptomycin and IFNγ (10 U/ml) at 33°C with 5% CO_2_ on type 1 collagen coated flasks as previously described [21]. Immortalized human podocytes (gift from Moin Saleem, Bristol) were grown in RPMI-1640 cell medium (Biochrom) containing 10% FBS, penicillin/streptomycin and insulin-transferrin-sodium selenite (ITS) at 33°C with 5% CO_2_ as previously described [22]. Both podocytes cell lines were grown on plastic and incubated at 37°C with 5% CO_2_ for a minimum of 10 days to differentiate.

### Protein Extraction and Western Blot Procedure

Protein from differentiated podocytes was extracted in lysis buffer (RIPA Buffer: 0.6 g Tris in 100 mL ddH_2_O, pH 7.5, 0.88 g NaCl, 0.1 g SDS, 0.5 g NaDeoxycloate, 1.0 g NP-40 Tergitol), 100 µl 100 mM NaVO_4_, 500µl 1 M NaF, and 1 Complete Mini Protease Inhibitor Cocktail Tablet (Roche Scientific) combined with okadaic acid). The podocyte extracts were frozen at −80°C for 1 hour, centrifuged at 4°C for 15 minutes at 11,000 rpm and the supernatant was recovered. Protein concentration was determined with the Bicinchoninic Acid (BCA) Protein Assay using the Sunrise TECAN plate reader and XRead Plus Software. SDS-PAGE and Western analysis were performed on 10 µg or 20 µg of these podocyte extracts. Western blots were probed with primary antibodies, p-cofilin (1∶1000) (Santa Cruz) or cofilin-1 (1∶5000) (Cytoskeleton) followed by the HRP labeled goat anti-rabbit secondary antibody (1∶10,000) (Santa Cruz). The PIERCE Chemiluminesence kit was used to detect the HRP labeled antibodies with Celtic SMR Ltd Cronex 5 film. The samples were normalized using GAPDH primary antibody (Santa Cruz).

For time course stimulations, cells were incubated in starvation media (supplemented RPMI media except with 1% FBS overnight at 37°C). The following morning media containing the stimulating agents, PMA (2µM) and TGF-β (5 ng/ml), was added to the cells. At each desired time point, cells were harvested and protein extractions were prepared.

### Quantitative PCR analysis

Total RNA was prepared from human glomeruli and cultivated human or murine podocytes using the RNeasy®MiniKit (Qiagen, Hilden, Germany) following the protocol recommended by the manufacturer with an additional step of DNAse digestion (RNase-Free DNase Set; Qiagen). One microgram of total RNA was reverse transcribed using Oligo (dT) 15 and random primers and M-MLV Reverse Transcriptase (Promega, Mannheim, Germany). Quantitative PCR (qPCR) was performed on a Light Cycler 480 (Roche, Mannheim, Germany). The cDNA was amplified using Fast Start Taq Polymerase (Roche Diagnostics, Mannheim Germany), SYBR Green (Molecular probes, Eugene, OR), gene specific primers and the following PCR conditions: 5 min at 95°C, 45 cycles for 10 s at 95°C, 10 s at 60°C and 10 s at 72°C. Specificity of the amplification product was verified by melting curve analysis. The samples were measured as multiplexed reactions and normalized to the constitutive gene human Hypoxanthin-Phosphoribosyl Transferase 1 (HPRT-1).

All primers for the listed transcripts were designed using Primer3 software:

mADF left: CGA AGG ATG CCA TCA AG; mADF right: GGT CCG ATT GAG GTC TTC; hADF left: CTA TGC AAG CTC CAA GGA; hADF right: CTG GTC CAT TTG CTT GAC; mCofilin-1 left: CAA GGA TGC CAT CAA GAA; mCofilin-1 right: GTC CTT GAC CTC CTC GTA; hCofilin-1 left: TTT ATG CCA GCT CCA AGG AC; hCofilin-1 right: GCT TGA TCC CTG TCA GCT TC; mCofilin-2 left: TGC TAG CTC TAA AGA TGC CAT T; mCofilin-2 right: AGC CAT TTA ACT TGC CAC TCA; hCofilin-2 left: TGC TAG CTC TAA AGA TGC CAT T; hCofilin-2 right: CAA GCC ATT TAC TTG CCA CTC.

### Histology

Paraffin embedded murine and human tissue slides were deparaffinized following a standard protocol. After washing and blocking, the tissues were incubated in primary antibodies to cofilin-1 (1∶25), p-cofilin (1∶100) and synaptopodin antibody (undiluted tissue culture supernatant) or podocalyxin antibody (1∶50). The tissues were then incubated either with Cy3-labeled donkey anti-rabbit or FITC-labeled goat anti-mouse or Alexa-488 labeled donkey anti- rabbit secondary antibodies. Nuclei were visualized using a DAPI counter stain. Images were taken using a Zeiss Axioplan-2 imaging microscope with the digital image-processing program AxioVision 4.3 (Zeiss, Jena, Germany).

### Cofilin-1 Silencing

Murine podocytes were grown overnight at 33°C with IFNγ. The following day RPMI-1640 media without penicillin/streptomycin was added. The podocytes were transfected with cofilin-1 and control siRNA (Santa Cruz Biotechnology Inc., Santa Cruz, CA), using Lipofectamine 2000 Reagent (Invitrogen). The cofilin-1 specific duplex siRNAs purchased from Santa Cruz contain a pool of 3 target-specific 19–25 nt siRNAs designed to knock down gene expression. After silencing, the podocytes were grown for 72 hrs in regular medium under non-permissive conditions at 37°C. Cytochalasin D (20µM) was applied to disrupt the actin network organization for 30 minutes at 37°C. After several washes with PBS, the cells were recovered in RPMI media for 0, 1 hr, 2 hr and 8 hr time points. The podocytes then were fixed, blocked and stained with FITC-labeled phalloidin and DAPI and mounted for microscopy. For the cell wound-healing assay, the cells were grown to confluency and a wound was applied using a sterile pipette tip. Images were taken of identical regions after wounding and 24 hours later. The wound area was quantified using ImageJ Software.

### Actin polymerization assay

The Polymerization Biochem Kit was obtained from Cytoskeleton, Inc. (Denver, CO). The podocytes were lyzed in lysis buffer (50 mM Tris (pH 7,5), 150 mM NaCl, 0,1% SDS, 0,5% Na-Deoxycholate, 1% Nonidet p-40/tergitol). 30 µg cell extract was mixed with actin polymerization buffer (50 mM KCl, 10 mM Tris (pH 7.5), 1 mM MgCl_2_, 1 mM ATP). After addition of pyrene-labelled actin (0.5 µM), fluorescence was measured every 30 seconds at 22°C in a spectrophotometer (excitation wavelength: 365 nm and emission wavelength: 407 nm).

## Results

### Loss of cofilin-1 induced foot process effacement and proteinuria in zebrafish

**Figure 1:**
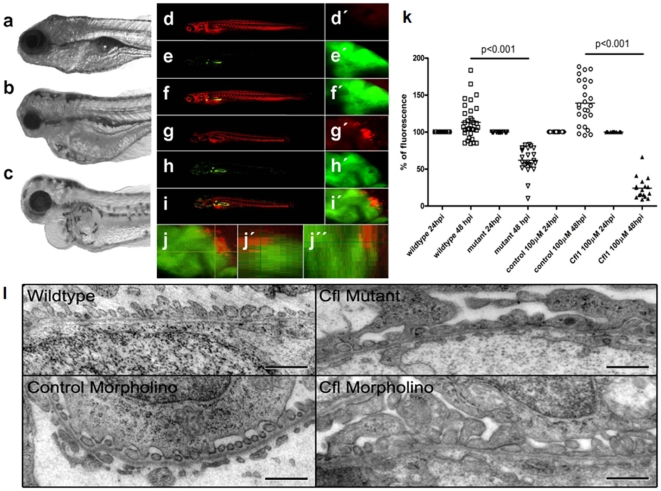
Knockdown of cofilin-1 expression by both morpholino silencing and viral insertional mutagenesis resulted in altered zebrafish phenotypes, compromised glomerular filtration and podocyte effacement. The phenotype of mutant (b) and morphant (c) embryos were compared to wildtype zebrafish embryos at 120 hpf (a). The mutant and morphant embryos had several aberrant features including edema around the yolk, pericardial effusion, smaller eyes, an arched back, jaw malformation and an absent swim bladder. Control Wt1b-GFP transgenic zebrafish (d–f, d′–f′) and those treated with cofilin-1 morpholinos (g–i, g′–i′) were injected with 70 kDa rhodamine labeled dextrans at 72 hpf and their pronephros imaged 48 hours later. The rhodamine labeled dextrans remained within the circulatory system of the Wt1b-GFP zebrafish with no labeling within the Wt1b-GFP pronephros tubules (d–f, d′–f′). In contrast, in the Wt1b-GFP transgenic morphant embryos (g–i, g′–i′), the vasculature was labeled by the rhodamine labeled dextran, but labeling was also observed within the pronephros tubules (magnification  = 180×). (j–j″) XZ cross section of the collected optical stacks clearly demonstrated the intracellular localization of rhodamine labeled dextrans within the pronephros tubules (yellow). (k) Functional studies demonstrated loss of pronephros function in cofilin-1 knockdown zebrafish embryos. Images of intravascular fluorescence were captured in retinal blood vessels and monitored in individual fish over 24 hours in control, cofilin-1 mutant zebrafish and zebrafish treated with 100µM cofilin-1 morpholinos. At forty-eight hours post injection of FITC-labeled 70 kDa dextrans into the vascular system, the fluorescence intensity in the pupil of the zebrafish increased in wildtype embryos, but decreased in zebrafish treated with 100 µM cofilin-1 morpholinos and cofilin-1 mutants. (l) TEM analysis of podocytes in control, cofilin-1 mutant and cofilin-1 morpholino treated zebrafish demonstrated severe podocyte effacement in cofilin-1 knockdown embryos. While wildtype (upper left) and control morpholino injected (lower left) zebrafish embryos have normal foot processes and slit diaphragm formation, the cofilin-1 mutants (upper right) as well as the cofilin-1 morpholino injected fish (lower right) display foot process fusion and foot process effacement over large areas (size bar: 500 nm).

To test the effects of cofilin-1 deficiency *in vivo*, zebrafish cofilin-1 mutants and morpholino induced knockdowns were analyzed. We found mutation of the cofilin-1 gene or knockdown of cofilin-1 expression resulted in severe edema in larval zebrafish. Mutant (Fig. 1b) and morphant (Fig. 1c) zebrafish, reared to 120 hour post fertilization showed consistent phenotypes. They both display edema around the yolk, pericardial effusion, no swim bladder, smaller eyes, arched backs and altered jaw features compared to the wildtype embryos (Fig. 1a). On Western blots we confirmed that cofilin-1 protein was significantly down regulated in morpholino injected fish (data not shown). To test whether the edematous phenotype was based on a disturbance of the glomerular filtration barrier, we applied different functional assays in live zebrafish embryos to examine leakiness of the filter to high molecular weight proteins. In figure 1, panels d–j, we used the Wt1b transgenic zebrafish [23] that displays green fluorescence in the fused glomeruli as well as in the proximal tubules of the pronephros (Fig. 1, panel e and h). We injected fertilized eggs with control (Fig. 1, panels d–f) and cofilin-1 morpholinos (Fig. 1, panels g–i) to produce morphants that expressed GFP in the pronephros. These morphants and controls were then injected with a 70 kDa rhodamine labeled dextran at 72 hpf into the cardinal vein (Fig. 1, panels d and g). After injection we imaged the pronephros 48 hours later (see merged images Fig. 1, g/g′ and i/i′). In Wt1b control embryos, no rhodamine labeled dextran was visible within the GFP-labeled proximal tubule region of the pronephros (Fig. 1, panel f′). In contrast, the morphant pronephros was leaky for the high molecular weight dextran and the rhodamine labeled dextran located within the Wt1b-GFP tagged proximal tubular cells of the morphants (Fig. 1, panel i′). In panels j, j′ and j″ the XZ reconstruction of the proximal tubules with localization of rhodamine labeled dextran within the GFP labeled tubular cells is depicted. These studies demonstrated exemplarily in a morphant fish that cofilin-1 deficiency led to disruption of barrier function of the glomerular filter. To demonstrate and reproduce these studies in a larger variety of embryos and to include the mutant in the analysis, we applied a different functional test. We measured loss of intravascular fluorescence activity using an eye assay that we established previously [18]. This assay enables us to measure leakiness of the glomerular filtration barrier as a rapid screening tool in individual morphant and mutant fish. When we monitored FITC-fluorescence activity in retinal blood vessels of individual zebrafish, we found stable or increasing fluorescence activity over a 24 hours period in wildtype or control morpholinos injected embryos (wildtype mean  = 113%±23, n = 33; control morpholino mean  = 139%±29, n = 25). Some embryos even showed an increase in fluorescence activity due to better intravascular distribution over time, in contrast, zebrafish injected with cofilin-1 morpholinos and cofilin mutants exhibited a significant decrease in FITC fluorescence over 24 hours (cofilin morpholino mean  = 24%±14, n = 17; cofilin mutant mean  = 62%±17, n = 24; p<0.001 control and wildtype vs. morpholino and mutant) (Fig. 1, panel k). To determine if the structural integrity of podocytes was compromised in the cofilin-1 morphant and mutant embryos, we analyzed glomeruli of 120 hpf embryos by transmission electron microscopy. Podocyte effacement was observed in both cofilin-1 morphant and mutant fishes whereas in control fish podocyte foot processes and slit diaphragms were clearly visible (Fig. 1, panel l). These data indicate that cofilin-1 is an indispensable factor for the integrity of normal podocyte foot processes in zebrafish.

### Cofilin-1 is expressed in murine and human podocytes

**Figure 2:**
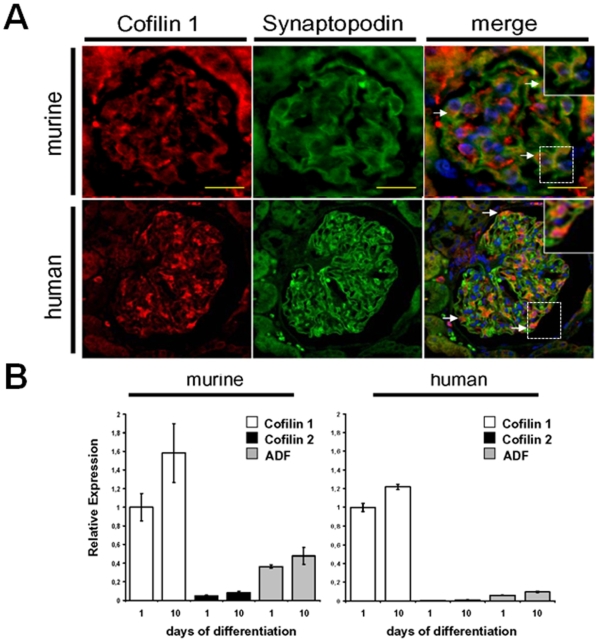
Cofilin-1 is the dominant ADF/cofilin family isoform in murine and human podocytes. (A) Using a primary rabbit antibody to cofilin, a diffuse distribution of cofilin throughout the cytoplasm was observed in both murine and human control podocytes (2A, red). In wildtype murine and normal human glomeruli, expression of cofilin colocalized with synaptopodin, a podocyte specific marker (2A, merge white arrows, insert enlarged 10×). (B) Analysis of RNA lysates of conditionally immortalized murine and human podocytes demonstrated cofilin-1 is the dominant isoform compared to cofilin-2 and ADF expression levels. The concentration of the cofilin-1 message is approximately 10 times higher than cofilin-2 and 3 times higher than ADF in murine podocytes and approximately 10 times higher than ADF in human podocytes, whereas cofilin-2 mRNA is barely detectable in human podocytes. Murine and human podocyte ADF/cofilin isoform expression levels did not significantly differ between one and ten days of differentiation.

Normal murine and human kidney tissues were probed with antibodies to cofilin and synaptopodin, a podocyte specific marker, to demonstrate cofilin-1 localized to podocytes *in vivo*. These studies confirmed localization of cofilin-1 along with synaptopodin in murine and human podocytes (Fig. 2A, white arrowheads). In murine tissue slides, cofilin-1 expression was also visible in synaptopodin negative glomerular cells, whereas cofilin-1 expression was primarily restricted to podocytes in human glomeruli.

Using Q-PCR experiments, we examined undifferentiated and differentiated murine and human cultured podocytes to determine the expression levels of the actin depolymerizing factor/cofilin (AC) family. We determined cofilin-1 mRNA was expressed at least three times more than the ADF-isoform in murine podocytes and ten times more than ADF in human podocytes (Fig. 2B). As expected, expression of the muscle specific isoform cofilin-2 was negligible in both podocyte species. When we compared undifferentiated (proliferating) and differentiated (non-proliferating) murine and human podocytes, we found expression of cofilin-1 and ADF was only slightly increased in both species at the differentiated state. These results suggested the predominant AC-isoform in podocytes is cofilin-1.

### Cofilin-1 activity is altered in response to TGF-β and PMA *in vitro*

**Figure 3:**
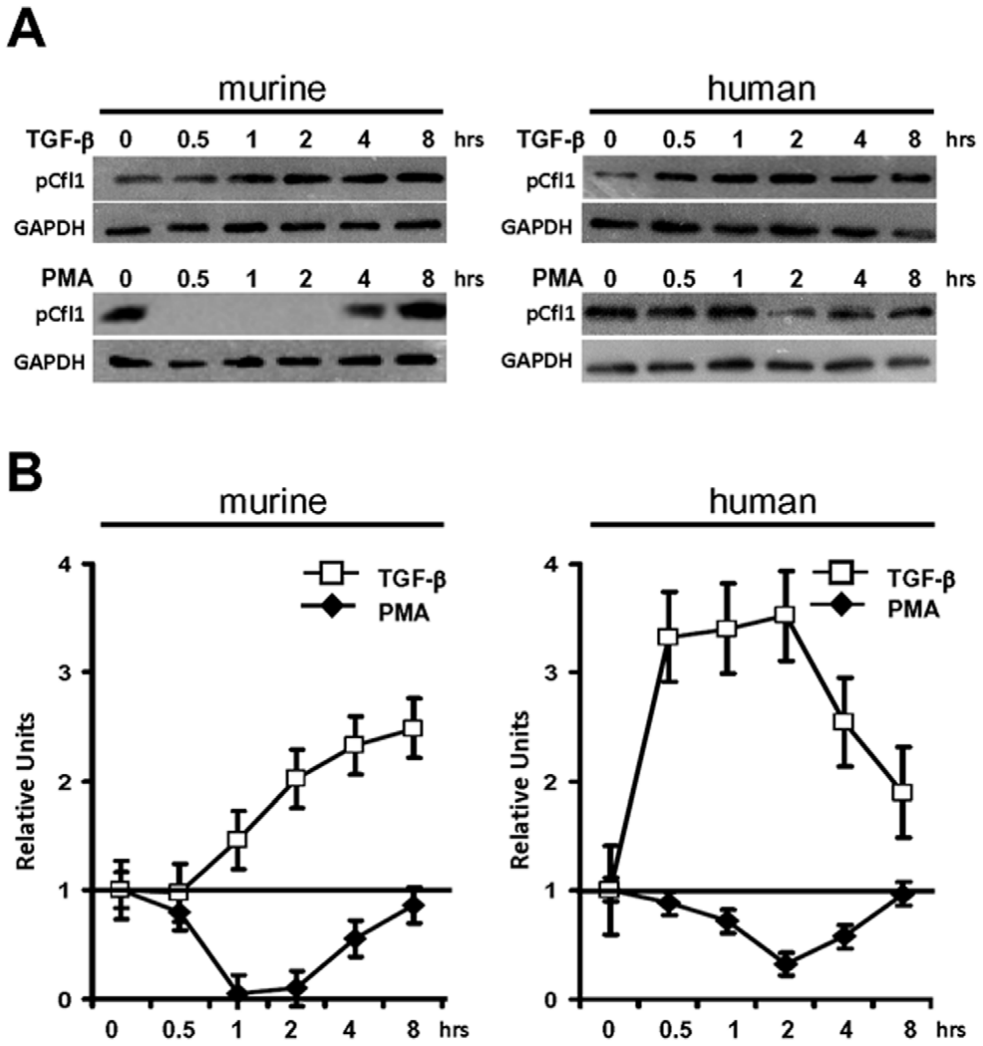
Treatment of cultured podocytes with PMA and TGF-β regulated cofilin-1 phosphorylation and activity. (A) Western blot analysis using the p-cofilin specific antibody demonstrated cofilin-1 regulation through serine 3 phosphorylation in murine and human podocytes treated with PMA and TGF-β. Phosphorylation profiles in response to 8 hours of treatment with TGF-β demonstrated a very strong increase in cofilin-1 phosphorylation resulting in cofilin-1 inactivation in murine and human podocytes. In contrast treatment with PMA led to dephosphorylation and activation of cofilin-1 in murine and human podocytes. GAPDH was probed as a protein loading control. (B) Densitometric summary of p-cofilin/GAPDH ratios of three independent experiments.

To determine the regulation of cofilin-1 activity in podocytes, we analyzed phosphorylation profiles in differentiated murine and human cultured podocytes *in vitro* in response to different stimuli. Interestingly, we could already detect baseline phosphorylation of cofilin-1 in both species (Fig. 3A). When we analyzed cofilin-1 phosphorylation in response to the known podocyte stressor, TGF-β, we found that in both murine and human species TGF-β stimulation led to increased cofilin-1 phosphorylation and reduced cofilin-1 activity. In contrast, when we stimulated podocytes with phorbol 12-myristate 13-acetate (PMA), which is often used as an activating phorbol ester of protein kinase C (PKC), we could document a significant dephosphorylation of cofilin-1 in both murine and human podocytes (Fig. 3A and B). This is consistant with several reports describing PKC activation as a known regulator of actin cytoskeletal dynamics in various cell types [24]. These data indicate that cofilin-1 phosphorylation can be regulated in podocytes in response to extracellular stimuli in both directions leading to an increase or decrease of cofilin-1 activity.

### Silencing of cofilin-1 leads to defects in actin filament disassembly and reduced migration activity in podocytes

**Figure 4:**
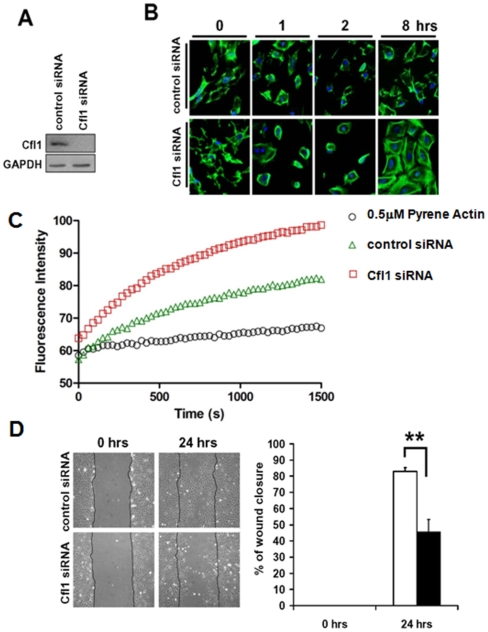
Murine podocytes transfected with cofilin-1 siRNA recovered more rapidly from cytochalasin D treatment than control cells and accumulated more actin, but have a reduced migration capability. (A) Western blot analysis of control and cofilin-1 siRNA transfected cell extracts demonstrated cofilin-1 protein expression was silenced by siRNA transfection. (B) Both control and cofilin-1 siRNA cells treated with cytochalasin were observed for recovery at 0, 1, 2 and 8 hours post treatment. At 0 and 1 hour time points, no discernible difference was observed in the control and cofilin-1 siRNA treated cells. By 2 hours post treatment, the cytochalasin effects on the cofilin-1 siRNA treated cells was diminished compared to the control siRNA cells. By 8 hours a significant difference in cell shape, size and cell spread was observed in the cofilin 1 siRNA treated cells compared to control cells. (C) Actin polymerization assay demonstrated an increased accumulation of actin fibers in podocytes in the absence of cofilin-1 (red squares) compared to control silenced podocytes (green triangles). Pyrene Actin in the absence of cell lysates serves as background control (black circles). (D) Control and cofilin-1 siRNA treated podocytes grown to confluence were wounded (0 hours) and their recovery examined 24 hours later. Control siRNA podocytes migrated to fill in the wound region at a significantly faster rate than podocytes treated with cofilin-1 siRNA.

To establish cofilin-1 was integral to actin dynamics in podocytes, cofilin-1 expression was silenced with siRNA (Fig. 4A). When we stained for actin fibers under normal culture conditions, we did not observe any significant changes in actin morphology in the cofilin-1 silenced cells (data not shown). To determine if silencing of cofilin-1 expression affected actin cytoskeletal recovery, we treated control silenced cells and cells treated with cofilin-1 siRNA with cytochalasin D (Fig. 4B). When we sequentially recovered the cells and stained for actin fibers, we found accumulation of radial actin bundles in cofilin-1 silenced cells eight hours after treatment with cytochalasin D. These results suggested the presence of cofilin-1 enhanced actin depolymerization and led to decreased formation of actin stress fibers as a prerequisite for recovery and cellular spreading in culture. Moreover, when we performed actin polymerization assays, we found a significant enhancement of actin accumulation in the cofilin-1 silenced podocytes (Fig.4C). Both findings indicated a profound defect in actin filament disassembly. Without expression of cofilin-1, the podocytes although initially characterized by small cells with many extended filopodia compared to control cells, began to spread and showed evidence of forming normal actin stress fibers by 2 hours post cytochalasin D treatment. Control cells did not demonstrate these characteristics until eight hours post treatment. Since actin turnover is required for migration of cells we wanted to test whether silencing of cofilin-1 would interfere with podocyte migration behavior in culture. To examine podocyte migration, we administered scrape wounds to control and cofilin-1 silenced podocyte cultures. We determined cofilin-1 silenced podocytes migrated slower and covered the wounded area significantly slower than control silenced cells (Fig. 4D). These data indicated that cofilin-1 activity was required for normal actin filament turnover and cellular migration activity in podocytes.

### In diseases associated with foot process effacement, cofilin-1 was inactivated and translocated to the nucleus of podocytes

**Figure 5:**
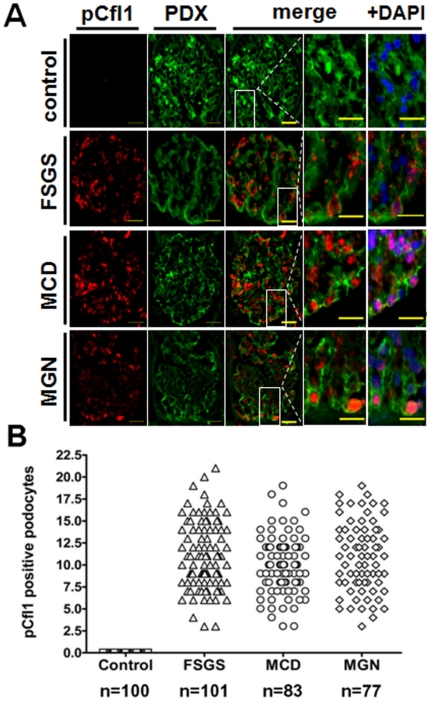
Phosphorylated cofilin-1 was increased in podocyte nuclei of patients suffering from FSGS, MCD and MGN. Glomeruli recovered from patients with glomerular diseases, FSGS, MCD and MGN, were probed with the rabbit primary antibody to p-cofilin and goat primary antibody to podocalyxin (PDX) and visualized using an Alexa488 labelled antibody raised against goat (green) and a Cy3-labelled secondary antibody against rabbit (red). Podocytes in these diseased glomerular tissues demonstrated an increased incidence of phosphorylated cofilin-1 in their nuclei (merged images ±DAPI). In contrast p-cofilin of normal control patients revealed no glomerular staining indicating that the cofilin-1 expressed in the glomerulus under normal conditions is active and under disease-conditions is inactivated.

The AC family of proteins is regulated by phosphorylation of serine 3. When serine 3 is phosphorylated, these proteins cannot bind globular or filamentous actin and are considered inactive. When serine 3 is dephosphorylated, these proteins are activated and bind both forms of actin [17]. Numerous studies have demonstrated that AC proteins are regulated by upstream stimuli in various cell types [14]. We examined 100 glomerular cross sections of five different human control samples and determined phosphorylated cofilin-1 was absent in healthy, control patients without proteinuria (Fig. 5A). Therefore, cofilin-1 is dephosphorylated and active under normal homeostasis conditions (Fig. 5B). In contrast, analysis of biopsies from patients with nephrotic glomerular disease (7 cases of FSGS, 6 MCD and 8 MGN) demonstrated p-cofilin-1 located to the nucleus in the majority of podocytes. In the FSGS cases, the average nuclear p-cofilin expression was 11±1.62 (range: 3–21 positive podocyte nuclei, n = 101 examined glomerular cross sections). In MCD the average p-cofilin-1 expression was 10±0.72 (range: 3–19, n = 83) and in MGN the average p-cofilin expression was 11±1.47 (range: 3–19, n = 77) (Fig. 5B). In summary, we did not observe a single glomerulus in the normal state that contained podocytes with inactive p-cofilin in their nucleus while in the diseased states we did not observe a single glomerulus with podocytes negative for inactive cofilin. These results are consistent with the hypothesis that inactivation of cofilin-1 occurs in disease states in podocytes.

## Discussion

Podocyte foot processes are highly dynamic cell extensions with an actin-based contractile apparatus. Since the glomerular capillary pressure constantly changes with blood pressure, it is likely that the foot processes experience distension of the capillary wall. Therefore, podocytes must be able to adapt to these changes to assure a network of functional filtration slits [6], [25]. In this study we examined the role of cofilin-1, a member of the actin depolymerizing factor/cofilin family of proteins, in regulation of F-actin assembly and turnover in podocytes. These studies demonstrated cofilin-1 plays an essential role in normal functions of podocytes. Our findings are supported by the following lines of evidence. We demonstrated for the first time, that cofilin-1 was required for the integrity of the podocyte filtration barrier *in vivo* in two models of cofilin-1 deficiency. The cofilin-1 mutant was generated via a proviral insertion into the cofilin-1 gene. This phenotype could be reproduced using a knockdown of cofilin-1 with morpholino techniques. In both systems we demonstrated functionally and morphologically that without cofilin-1 normal foot processes are not present. Instead the cofilin-1 knockdown and the cofilin-1 mutation results in the typical effacement phenotype without formation of secondary podocyte processes similar to the podocyte effacement phenotype observed in human disease states. We showed in murine and human cells in culture, that cofilin-1 was the dominant isoform of the AC family of proteins in podocytes and we clearly demonstrated cofilin-1 expression along with the podocyte specific marker synaptopodin in murine and human tissues. This is well in line with the findings of *Vartiainen et al*., who have demonstrated earlier that cofilin-1 and ADF are the most abundant isoforms in the kidney [28]. Interestingly, when we examined human control tissues for expression of inactivated, phosphorylated cofilin-1, we did not detect any phosphorylated cofilin-1. In contrast in disease states we detect a significant increase in p-cofilin expression. This indicates the presence of inactive cofilin-1 coincides with high level proteinuria and foot process effacement and could be interpreted as indirect evidence that active cofilin-1 is required for normal podocyte function.

When we exposed murine and human podocytes to TGF-β, we found that TGF-β stimulation led to an increase in cofilin-1 phosphorylation. This is particularly relevant since we and others have described TGF-β as a causative factor for disease initiation and progression of proteinuric diseases in mice and humans. TGF-β activation is a well described pathomechanism in various glomerular diseases with proinflammatory and profibrotic activity and has direct proapoptotic effects on podocytes [8], [26], [27]. Therefore, it is tempting to speculate that cofilin-1-inactivation is linked to proteinuria in different disease states with TGF-β activation. On the other hand, stimulation with PMA induces a strong activation response on cofilin-1. Moreover, baseline-phosphorylation of cofilin-1 was detectable in resting unstimulated podocytes in culture and cells can respond with more activation as well as more inactivation. We presume that the regulation of cofilin-1 activity is under autocrine control with the potential to respond with more or less activation depending on the cellular micromillieu and physiological context. Our data are supported by functional *in vitro* experiments where we can demonstrate that cofilin-1 activity is required for actin bundling and podocyte motility. Numerous studies link AC proteins to the formation of cellular protrusions due to cofilin's critical roles in accelerating ATP hydrolysis, increasing actin treadmilling, polymerization and depolymerization of actin filaments, and filament severing. Cellular protrusions, which are prerequisites for cell movement and subsequent cell migration, result from newly polymerized actin filaments pushing the cellular membrane forward. This process has been observed in the formation of filopodia, pseudopodia, lamellipodia and growth cones. These cellular extensions are dynamic and can be observed during normal development and morphogenesis, in the immune response of lymphocytes, in metastasis of cancer cells, neuronal growth cones and in response to wounding. Similar findings with diminished actin filament disassembly, decreased motility and increased amounts of actin fibers were published earlier in other mammalian cells [29] and even a defective migration of cells *in vivo* has been published earlier [30]. In our studies we demonstrated the essential role of active cofilin in podocyte cell migration. When cofilin was silenced in cultured podocytes with cofilin siRNA, the podocytes response to healing after wounding was severely delayed compared to the response of control cells to the wound. Since the cofilin silenced podocytes did not extend filopodia similar to the control cells, this suggested active cofilin was required for formation of normal filopodia extensions. Without active cofilin, the breakdown and formation of new actin filaments beneath of the plasma membrane would be severely limited and could no longer put forward pressure on the overlying membrane to protrude. Also, an intact actin cytoskeleton would be required for formation of focal contacts that are required to pull the cell forward. The requirement of active cofilin in migration has been previously demonstrated in Cos and MCF7 cells where lamellipodia extensions in response to growth factors were reduced when cofilin was inactivated [31] and in MRLn3 cells where maturation of invadopodia were suppressed when cofilin was inhibited [32].

Thus, we hypothesize that the expression of phosphorylated cofilin-1 in glomerular diseases suggests a reduced capacity of podocytes to adapt to glomerular pressure differences. A higher filtration pressure and distension of the capillary wall can not be compensated and leads to proteinuria. Since proteinuric patients are regularly treated with drugs reducing the glomerular filtration pressure, this mechanism could partially explain the success of these treatment regimens, which have been proven to reduce proteinuria and are considered podocyte protective [33]. Recently, *Garg et al.* described a podocyte specific mechanism of cofilin-1 activation. They demonstrated a nephrin induced cofilin dephosphorylation and found that the podocyte specific cofilin-1 knockout develops proteinuria [34].

In summary, we conclude that cofilin-1 is an essential regulator for recycling of actin-filaments in podocytes to ensure the dynamic nature of the foot processes. This could be the basis of a very common mechanism in human proteinuric diseases leading to the same phenotype, effacement and inability to form or maintain foot processes.

## References

[1] Tryggvason K, Patrakka J, Wartiovaara J (2006). Hereditary proteinuria syndromes and mechanisms of proteinuria.. N Engl J Med.

[2] Faul C, Asanuma K, Yanagida-Asanuma E, Kim K, Mundel P (2007). Actin up: regulation of podocyte structure and function by components of the actin cytoskeleton.. Trends Cell Biol.

[3] Kriz W, Lemley KV (1999). The role of the podocyte in glomerulosclerosis.. Curr Opin Nephrol Hypertens.

[4] Kerjaschki D (2001). Caught flat-footed: podocyte damage and the molecular bases of focal glomerulosclerosis.. J Clin Invest.

[5] Takeda T, McQuistan T, Orlando RA, Farquhar MG (2001). Loss of glomerular foot processes is associated with uncoupling of podocalyxin from the actin cytoskeleton.. J Clin Invest.

[6] Kriz W, Mundel P, Elger M (1994). The contractile apparatus of podocytes is arranged to counteract GBM expansion.. Contrib Nephrol.

[7] Schiffer M, Haller H (2006). [Focal segmental glomerulosclerosis (FSGS). Molecular defects and clinical relevance].. Dtsch Med Wochenschr.

[8] Schiffer M, Bitzer M, Roberts IS, Kopp JB, ten DP (2001). Apoptosis in podocytes induced by TGF-beta and Smad7.. J Clin Invest.

[9] Peters I, Tossidou I, Achenbach J, Woroniecki R, Mengel M (2006). IGF-binding protein-3 modulates TGF-beta/BMP-signaling in glomerular podocytes.. J Am Soc Nephrol.

[10] Kaplan JM, Kim SH, North KN, Rennke H, Correia LA (2000). Mutations in ACTN4, encoding alpha-actinin-4, cause familial focal segmental glomerulosclerosis.. Nat Genet.

[11] Weins A, Schlondorff JS, Nakamura F, Denker BM, Hartwig JH (2007). Disease-associated mutant alpha-actinin-4 reveals a mechanism for regulating its F-actin-binding affinity.. Proc Natl Acad Sci U S A.

[12] Weins A, Kenlan P, Herbert S, Le TC, Villegas I (2005). Mutational and Biological Analysis of alpha-actinin-4 in focal segmental glomerulosclerosis.. J Am Soc Nephrol.

[13] Bamburg JR (1999). Proteins of the ADF/cofilin family: essential regulators of actin dynamics.. Annu Rev Cell Dev Biol.

[14] Van TM, Huyck L, Leyman S, Dhaese S, Vandekerkhove J (2008). Ins and outs of ADF/cofilin activity and regulation.. Eur J Cell Biol.

[15] Scott RW, Olson MF (2007). LIM kinases: function, regulation and association with human disease.. J Mol Med.

[16] Huang TY, DerMardirossian C, Bokoch GM (2006). Cofilin phosphatases and regulation of actin dynamics.. Curr Opin Cell Biol.

[17] Wiggan O, Bernstein BW, Bamburg JR (2005). A phosphatase for cofilin to be HAD.. Nat Cell Biol.

[18] Hentschel DM, Mengel M, Boehme L, Liebsch F, Albertin C (2007). Rapid screening of glomerular slit diaphragm integrity in larval zebrafish.. Am J Physiol Renal Physiol.

[19] Bollig F, Mehringer R, Perner B, Hartung C, Schafer M (2006). Identification and comparative expression analysis of a second wt1 gene in zebrafish.. Dev Dyn.

[20] Amsterdam A, Nissen RM, Sun Z, Swindell EC, Farrington S (2004). Identification of 315 genes essential for early zebrafish development.. Proc Natl Acad Sci U S A.

[21] Mundel P, Kriz W (1996). Cell culture of podocytes.. Exp Nephrol.

[22] Saleem MA, O'Hare MJ, Reiser J, Coward RJ, Inward CD (2002). A conditionally immortalized human podocyte cell line demonstrating nephrin and podocin expression.. J Am Soc Nephrol.

[23] Perner B, Englert C, Bollig F (2007). The Wilms tumor genes wt1a and wt1b control different steps during formation of the zebrafish pronephros.. Dev Biol.

[24] Djafarzadeh S, Niggli V (1997). Signaling pathways involved in dephosphorylation and localization of the actin-binding protein cofilin in stimulated human neutrophils.. Exp Cell Res.

[25] Kriz W, Kretzler M, Provoost AP, Shirato I (1996). Stability and leakiness: opposing challenges to the glomerulus.. Kidney Int.

[26] Schiffer M, Mundel P, Shaw AS, Bottinger EP (2004). A novel role for the adaptor molecule CD2-associated protein in transforming growth factor-beta-induced apoptosis.. J Biol Chem.

[27] Schiffer M, Schiffer LE, Gupta A, Shaw AS, Roberts IS (2002). Inhibitory smads and tgf-Beta signaling in glomerular cells.. J Am Soc Nephrol.

[28] Vartiainen MK, Mustonen T, Mattila PK, Ojala PJ, Thesleff I (2002). The three mouse actin-depolymerizing factor/cofilins evolved to fulfill cell-type-specific requiremnets for actin dynamics.. Mol Biol Cell.

[29] Hotulainen P, Paunola E, Vartiainen MK, Lappalainen P (2005). Actin-depolymerizing factor and cofilin-1 play overlapping roles in promoting rapid F-actin depolymerization in mammalian nonmuscle cells.. Mol Biol Cell.

[30] Gurniak CB, Perlas E, Wittke W (2005). The actin-depolymerizing factor n-cofilin is essential for neural tube morphogenesis and neural crest cell migration.. Dev Biol Cell.

[31] Kiuchi T, Ohashi K, Kurita S, Mizuno K (2007). Cofilin promotes stimulus-induced lamellipodium formation by generating an abundant supply of actin monomers.. J Cell Biol.

[32] Yamaguchi H, Lorenz M, Kempiak S, Sarmiento C, Coniglio S (2005). Molecular mechanisms of invadopodium formation: the role of the N-WASP-Arp2/3 complex pathway and cofilin.. J Cell Biol.

[33] Kriz W (2004). Podocytes as a target for treatment with ACE inhibitors and/or angiotensin-receptor blockers.. Kidney Int.

[34] Garg P, Verma R, Cook L, Soofi A, Venkatareddy M (2010). Actin-depolymerizing factor cofilin-1 is necessary in maintaining mature podocyte architecture:. J Biol Chem.

